# Synthesis, Phase-Transition Behaviour, and Oil Adsorption Performance of Porous Poly(*oligo*(ethylene glycol) Alkyl Ether Acrylate) Gels

**DOI:** 10.3390/polym12061405

**Published:** 2020-06-23

**Authors:** Syed Ragib Safi, Taku Nakata, Shyotaro Hara, Takehiko Gotoh, Takashi Iizawa, Satoshi Nakai

**Affiliations:** Department of Chemical Engineering, Hiroshima University, 1-4-1 Kagamiyama, Higashi Hiroshima, Hiroshima 739-8527, Japan; d196289@hiroshima-u.ac.jp (S.R.S.); m164382@hiroshima-u.ac.jp (T.N.); m144357@hiroshima-u.ac.jp (S.H.); tiizawa@hiroshima-u.ac.jp (T.I.); sn4247621@hiroshima-u.ac.jp (S.N.)

**Keywords:** thermosensitive polymer gel, phase-transition behaviour, porous gel

## Abstract

To probe the effects of pendant side-chain structures on the properties of porous thermoresponsive polymer gels, *oligo*(ethylene glycol) alkyl ether acrylates were polymerised in an aqueous medium under radical-mediated phase-separation conditions. The monomer structures varied according to the lengths and termini of their ethylene glycol side chains. The porous poly(*oligo*(ethylene glycol) alkyl ether acrylate) (POEGA) gels exhibited variable lower critical solution temperatures (LCSTs) but similar and rapid swelling–deswelling behaviours. Although the LCST of the poly(tri(ethylene glycol) monomethyl ether acrylate) (PTEGA) gel decreased with increasing aqueous NaCl or CaCl_2_ concentration, PTEGA showed excellent thermosensitivity in highly concentrated salt solutions, recommending its application in saline environments. Examination of PTEGA adsorption performance in an oil–water emulsion showed that *n*-tridecane adsorption increased with temperature. Although *n*-tridecane was effectively adsorbed at 70 °C, its release from the fully adsorbed PTEGA gel was difficult despite a temperature reduction from 70 to 20 °C.

## 1. Introduction 

Thermosensitive polymers have attracted research attention in fields as widespread as pharmaceutical drug delivery to engineering since the 1950s [1]. These materials show unique thermal behaviours in solution. Poly(*N*-isopropylacrylamide) (PNIPA), for example, undergoes solubility-decreasing structural reordering upon heating in an aqueous solution [1,2]; the temperature at which this occurs is known as the lower critical solution temperature (LCST), or the cloud point. Recently, poly[*oligo*(ethylene glycol) methyl ether acrylate) (POEGA) and poly(*oligo*(ethylene glycol) methyl ether methacrylate) (POEGMA) have gained attention as thermosensitive polymers that could potentially replace the widely used PNIPA [2,3,4,5] because of their biocompatible acrylate or methacrylate main chains and pendant *oligo*(ethylene glycol) side chains. The biggest advantage of these linear polymers and gels, easily obtained from the radical polymerisation of the corresponding monomers, is the manipulability of their LCSTs by controlling the length of the *oligo*(ethylene glycol) chain without adding co-monomers [2,4]. Further, the LCSTs can be precisely adjusted through the random copolymerisation of either *oligo*(ethylene glycol) methyl ether acrylate (OEGA) monomers [6] or *oligo*(ethylene glycol) methyl ether methacrylate (OEGMA) monomers [7] with different chain lengths. 

Side chains can impart unique responsiveness aside from thermosensitivity in *oligo*(ethylene glycol)-containing polymers and gels. The cloud points of poly(ethylene oxide) (PEO)–salt–water systems are known to decrease with increasing salt concentration, as PEO and its analogues easily form complexes with salts [8]. Lutz et al. [9] reported that the cloud points of linear POEGMA_300_ and its copolymers, as measured by the turbidimetric method in phosphate-buffered saline solution (essentially dilute salt water), are 3–4 °C lower than those in pure water. Magnusson et al. [10] reported that the cloud points of copolymers of *oligo*(ethylene glycol) ethyl ether methacrylate (*M_n_* = 246 g mol^−1^) and OEGMA (*M_n_* = 475 g mol^−1^) were ion-sensitive in water. However, because these gels are insoluble in any solvent, their detailed phase-transition behaviours cannot be estimated by the turbidimetric method.

Thermosensitive gels and their swelling behaviours in solvents can also be affected by the nature of their side chains [11]. This is exemplified by POEGA and POEGMA thermosensitive gels. However, because the swelling process is diffusion-limited, the swelling and deswelling of nonporous gels are too slow for practical use. To address this problem, porous gels and regular comb-like gels can be prepared, using thermosensitive side chains with narrow molecular-weight distributions. Comb-like gels have been prepared by polymerisation of the corresponding macromers [12,13] and the atom-transfer radical polymerisation of OEGMA using macroinitiator gels containing pendant 2-bromoisobutyrate groups [14]. Porous PNIPA gels have been prepared by several methods, including the incorporation of surfactants [15] or silica particles [16] during gel preparation and their subsequent extraction; thermal phase-separation polymerisation at temperatures above the LCST [17,18,19,20]; and freeze-drying [21]. In addition, porous copolymer gels of 2-hydroxyethyl methacrylate with OEGMA (*M_n_* = ~1100 g mol^−1^) have been prepared by photoinitiated phase-separation polymerisation in water or aqueous NaCl solutions [22]. Thermal phase-separation polymerisation is simpler and more economical than other methods. This approach has been applied to synthesise high-performance porous PNIPA gel products, including PNIPA gel plates reinforced with stainless-steel wire netting for improved mechanical characteristics [23,24,25] and monodisperse millimetre-order gel beads by sedimentation polymerisation [20,26].

In a previous communication [11], we reported the synthesis of porous POEGMA_300_ gels by the thermal phase-separation polymerisation of OEGMA_300_ (*M_n_* = ~300 g mol^−1^); these gels exhibited rapid swelling–deswelling and high thermosensitivity in a highly concentrated aqueous NaCl solution, although the LCSTs measured by the equilibrium swelling ratio were decreased with increasing NaCl concentration. No other thermosensitive gels are known to swell in highly concentrated aqueous NaCl solutions. The systematic phase-transition behaviours of POEGA and POEGMA gels have not been reported, although the turbidimetrically determined LCSTs of the corresponding linear polymers [2,4,5] are known.

**Figure 1:**
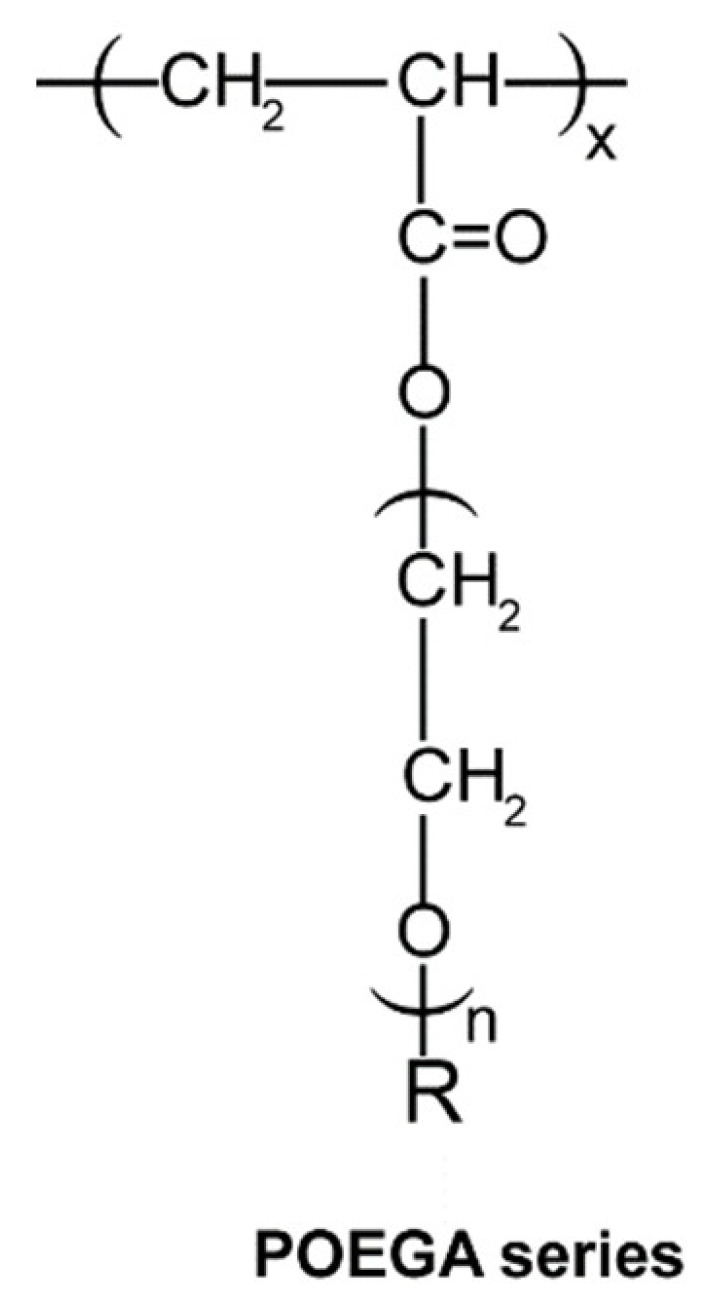
Structure of Poly(*oligo*(ethylene glycol) Alkyl Ether Acrylate) gel series (R = Me or Et; *n* = 2, 3, or 8–9).

This paper reports the preparation via thermal phase-separation polymerisation of a series of porous cylindrical POEGA gels with systematically varied side-chain substituents (Figure 1). Their phase-transition behaviours were evaluated based on their equilibrium external radius ratios of the fully swollen to shrunk gels as a function of temperature in pure water and aqueous NaCl or CaCl_2_ solutions. 

Various studies have investigated the separation of oil from oil–water emulsions. Tested techniques have included gravity separation [27], coagulation [28], electrochemical treatment [29], in situ burning [30], air flotation [31], filtration, and adsorption [32]. Most of these techniques have drawbacks such as low efficiency, high operational cost, generation of toxic by-products, and inability to reuse or recycle materials [29]. Adsorption avoids these issues; hence, adsorption technology [33] and membrane technology [34] are the mostly commonly used techniques for oil separation [35]. 

The hydrophobic surface of a membrane can be fouled by oil droplets, decreasing the efficiency of the membrane [36]. Consequently, the efficiency of membranes in separating oil from an oil–water emulsion has not been studied extensively [37]. Therefore, adsorption remains the established method for oil–water separation [35,38]. In addition to characterising the properties of POEGA gels, we have examined the oil adsorption performance of one gel specimen. 

## 2. Methods

### 2.1. Materials

Di(ethylene glycol) monomethyl ether acrylate (DEGA), di(ethylene glycol) monoethyl ether acrylate (eDEGA), tri(ethylene glycol) monomethyl ether acrylate (TEGA), *oligo*(ethylene glycol) monomethyl ether acrylate (OEGA_480_, *M_n_* = ~480 g mol^−1^), and di(ethylene glycol) diacrylate (DEGDA) were supplied by Hitachi Chemical Co. Ltd., Tokyo, Japan. The initiators, potassium peroxodisulfate (K_2_S_2_O_8_) and anhydrous sodium sulphite (Na_2_SO_3_), were purchased from Katayama Chemical Co. Ltd., Osaka, Japan and Wako Pure Chemical Industries Ltd., Osaka, Japan, respectively. The monomers and initiators were used without further purification. Tween 20 surfactant (polyoxyethylene sorbitan monolaurate), sodium chloride (NaCl), and ethanol (99.5%) were purchased from Nacalai Tesque, Inc., Kyoto, Japan. The oil adsorbate, *n*-tridecane (C_13_H_28_), was purchased from Tokyo Chemical Industry Co. Ltd., Tokyo, Japan. The solvents used in all experiments were purified by distillation. Prior to use, distilled water was sonicated for 30 min to release dissolved gases. Activated carbon (AC; activated charcoal granules) was purchased from Kanto Chemical Co. Inc., Tokyo, Japan. Results were compared against previously prepared porous and nonporous PNIPA gels [11]. 

### 2.2. Preparation of Thermosensitive Porous Gels

A typical synthesis of a porous gel is thus described. Tri(ethylene glycol) monomethyl ether acrylate (5.0 g, 23 mmol), DEGDA (98 mg, 0.46 mmol), and potassium peroxodisulfate (31 mg, 0.115 mmol) were dissolved in water (12 mL). The solution was charged in a 50 mL cylindrical flask containing approximately 25 Teflon tubes (internal diameter: 4 mm; length: ~3 cm; the tubes served as in situ polymerisation forms). To the flask was added a solution of anhydrous sodium sulphite (29 mg, 0.23 mmol) in water (3 mL) under a flow of nitrogen. The reaction mixture was stirred for 10 min and left to stand for 12 h at 80 °C to complete the gel polymerisation. After completion of gelation, the polymer-filled tubes were retrieved from the bulk gel in the flask. The filled tubes were sliced crosswise to produce cylindrical specimens of equal length and diameter. Uniformly shaped gel samples were obtained by pushing the gels from the tubes and immersing them in a large amount of methanol to wash away unreacted substances. The washing process was conducted for 24 h. The gels were then immersed in a large amount of water to remove the methanol. The water was replaced three times every 24 h. The resulting white sponge-like PTEGA pellets were placed in water and stored in a swollen condition in a refrigerator at 5 °C. The gels prepared by this procedure are summarised in Table 1. 

### 2.3. Measurement of Swelling Rates and Equilibrium External Radius Ratios

The swelling rates and equilibrium swelling ratios [22] of the pellets were measured as follows. A pellet was removed from the refrigerated storage and de-swelled by immersion in 80 °C water for 20 min. The retrieved pellet was placed in water at 10 °C and allowed to swell with periodic measurement of its external radius using a digital camera (Pentax Q7) until no further change was observed. The swelling rate is given by the change in the external radius ratio (*R_t_*/*R_0_*), where *R*_0_ and *R_t_* are the external radii of the de-swollen gel at 80 °C and the gel swollen in 10 °C water for *t* min, respectively. The equilibrium external radius ratios (*R_T_/R_0_*) can also be considered with the cube of *R_T_*/*R*_0_. In these expressions, *R_T_* is the measured external radius of the gel fully swollen in water at a given temperature of *T* °C. The equilibrium swelling ratios at *T* °C correspond to (*R_T_*/*R_0_*)^3^ because the fine cylindrical shape is retained during swelling which occurs isotropically. For the POEGA_480_ gel, the external radius *R*_0_ of the gel shrunken in an aqueous 5 wt % NaCl solution at 80 °C was adopted.

### 2.4. Morphological Evaluation of PNIPA and POEGA Gels 

A gel pellet was dipped in ethanol at 30 °C. The swollen gel was cut into several slices, which were then soaked in liquid nitrogen. The frozen samples were quickly transferred to a sample bottle and freeze-dried in liquid nitrogen under vacuum. Scanning electron microscopy (SEM) photographs were taken of the prepared samples using a JSM-5600 (JEOL Ltd., Tokyo, Japan). [20].

### 2.5. Measurement of Oil Adsorption and Desorption Rates of the PTEGA Gel 

To prepare the oil–water emulsions, *n*-tridecane and water with varying amounts of Tween 20 were mixed using a homogeniser. The detailed compositions of the oil–water emulsions are shown in Table 2.

The gel pieces for adsorption were first shrunk by heating them in an oven at 80 °C for 30 min. Adsorption was performed by immersing small gel pieces (50 mg at 80 °C) in the oil–water emulsions (5 mL) and stirring at 150 rpm and 70 °C for 24 h. After the adsorption experiment, a sample (1 mL) was collected in a 10 mL flask. Then, hexane (8 mL) and *n*-tridecane (1 mL, 1000 ppm) were added to the flask. The flask was shaken for 1 h, producing visible layers of oil and water. A sample (1 mL) of oil was collected from the upper layer of the flask and used for gas chromatography/mass spectroscopic (GC-MS) analysis by HP 6890 GC and HP 5973 MSD (Agilent Technologies, Inc., CA, USA). The remaining amount of oil was obtained from the peaks observed in GC-MS analysis.

Oil adsorption from the oil–water emulsion was evaluated by shaking the sample bottles containing the emulsion solution (5.0 mL) and PTEGA gel (50 mg) at 70 °C for 15, 30, 60, 90 and 120 min using a shaker. Oil desorption from the fully adsorbed PTEGA gel was accomplished by sonication of the bottles at 20 °C for 30, 60, 90 and 120 min after the shaking of the bottles at 70 °C for 120 min. From each bottle, 1.0 mL of the solution was collected. The adsorbed and desorbed amounts of oil in the PTEGA gel under each condition were calculated from the oil content of the solution as determined by GC-MS analysis.

## 3. Results and Discussion

### 3.1. Synthesis and Transition Behaviour of Porous POEGA Gels

The phase-separation polymerisation of the aqueous OEGA monomer solution was performed in Teflon tubes (internal diameter: 8 mm) at 80 °C (Table 1). Highly porous sponge-like gels were easily obtained [11]. However, the polymerisation of OEGA monomers besides TEGA did not yield uniform sponge-like gels when pure water was used as a solvent. For effective phase-separation polymerisation yielding a sponge-like gel, the polymerisation solvent must satisfy the following two conditions: (1) the monomer must be soluble in the solvent, and (2) the resulting gel must be de-swollen in the solvent at the polymerisation temperature. With PeDEGA and PDEGA, 30 and 15 wt % aqueous ethanol solutions were used, respectively, because the monomers did not dissolve well in water. In contrast, for the POEGA_480_ gel, phase separation occurred imperfectly as the gel was excessively hydrophilic and swelled in water up to 80 °C. For this reason, a small amount of NaCl was added to the aqueous monomer solution to prevent swelling at 80 °C. When the appropriate aqueous solvent systems were used, the monomer solutions immediately became cloudy during the reaction because of polymer precipitation. The polymer precipitates agglutinated into white sponge-like gels. Cylindrical gel pellets of equal lengths and diameters were obtained by retrieving and cutting the gel bars from the Teflon tubes. 

**Figure 2:**
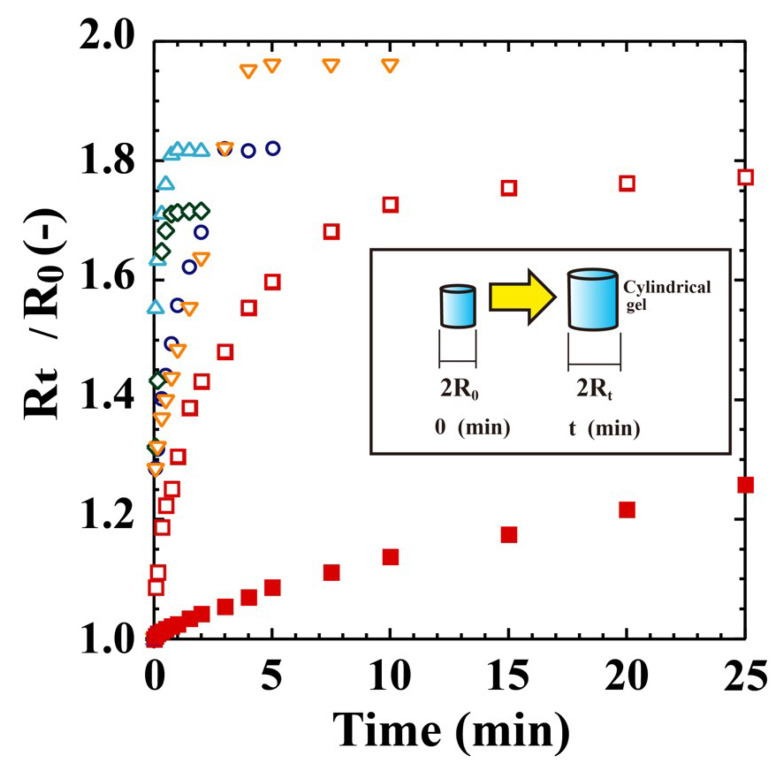
Swelling behaviours of POEGA gels in pure water at 10 °C. △: PeDEGA, ○: PDEGA, ◇: PTEGA, ▽: POEGA_480_, □: porous PNIPA, ■: nonporous PNIPA.

The swelling–deswelling properties of the POEGA pellets were investigated in water at 10 and 80 °C, respectively (Figure 2). For each hydrogel, swelling and deswelling were reversible, and the fine cylindrical shape was retained in water. The swollen gels shrank as soon as they were dipped in water at 80 °C; shrinking was much faster than swelling and could not be measured. Cracks were not detected during the swelling–deswelling cycles in the porous POEGA gels. These behaviours were the same as those observed previously for porous PNIPA and POEGMA gels [11]. 

The gels shrunk at 80 °C were next swollen in water at 10 °C. Figure 2 shows the external radius ratios (*R_T_*/*R*_0_) over time compared to those of porous and nonporous PNIPA gels. With the POEGA_480_ gel, the external radius *R*_0_ of the gel shrunken in aqueous 5 wt % NaCl solution at 80 °C was adopted because the gel swelled well in water at 80 °C. The nonporous PNIPA gel swells very slowly, requiring ~5 days to reach a point after which no further swelling is observed. In contrast, the porous PNIPA gel swells rapidly, reaching the maximum point in only 25 min. Notably, the PTEGA and PeDEGA gels achieve the point within 60 s. Therefore, the porous sponge-like POEGA gels exhibit a capacity for rapid swelling and deswelling, similar to the sponge-like POEGMA_300_ gel reported previously [11]. However, the swelling rate of the PTEGA gel was slightly higher than that of the POEGMA_300_ gel. 

**Figure 3:**
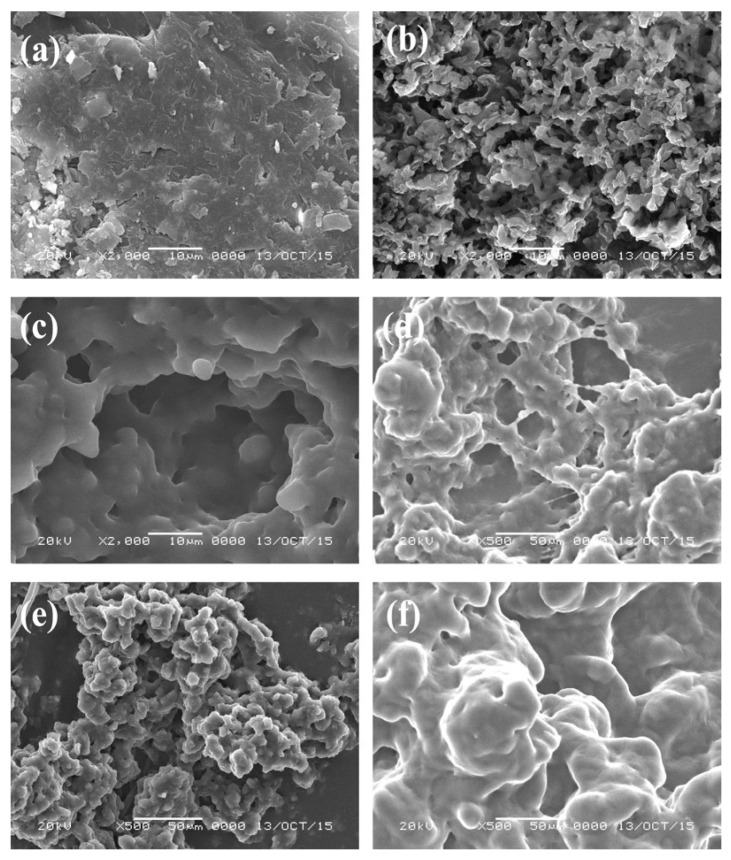
SEM images of (**a**) nonporous PNIPA gel; (**b**) porous PNIPA gel; (**c**) porous PeDEGA gel; (**d**) porous PDEGA gel; (**e**) porous PTEGA gel; and (**f**) porous POEGA_480_ gel (scale bar length: 10 µm).

The morphologies of the prepared PeDEGA, PDEGA, PTEGA, and POEGA_480_ gels were examined by SEM and were compared with those of porous and non-porous PNIPA gels [20] in Figure 3. The POEGA gels and porous PNIPA gel are highly porous with aggregated structures consisting of fine particles. The POEGA gels have larger pores than the porous PNIPA gel, although the latter has finer particles. These results support the observation of more rapid swelling and deswelling in the POEGA gels.

**Figure 4:**
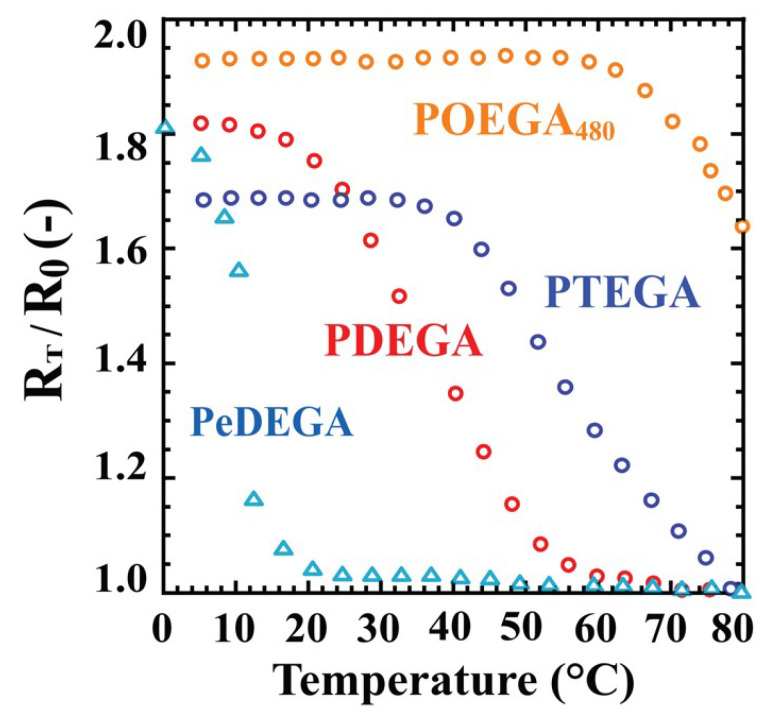
Transition behaviours of POEGA gels in pure water. △: PeDEGA, ○: PDEGA, ○: PTEGA, ○: POEGA_480_.

The equilibrium external radius ratios (*R_T_*/*R_0_*) for the POEGA gels as a function of temperature are shown in Figure 4. The equilibrium swelling ratios at *T* °C correspond to (*R_T_*/*R_0_*)^3^, because the fine cylindrical shape was retained during isotropic swelling in water. The equilibrium swelling ratios or equilibrium external radius ratios of the gels in water directly depend on their hydrophilicity. As the POEGA_480_ gel was well-swollen in water at 80 °C, the external radius *R_0_* of the gel shrunken in an aqueous 5 wt % NaCl solution at 80 °C was adopted. The figure indicates that the LCST increased with increasing length of the pendant *oligo*(ethylene glycol) groups. The ethyl-ether-terminated PeDEGA gel displayed a lower LCST than the methyl-ether-capped PDEGA gel. These results agree with those of the corresponding soluble linear polymers measured by the turbidimetric method [4]. However, the exact LCSTs of the gels were not fixed, because the POEGA gels did not show a significant temperature dependence for the external radius ratio. The LCSTs of the gels observed by the swelling method were slightly higher than the cloud points of the corresponding linear polymers. For example, the LCST of the PTEGA gel was ~80 °C, whereas the cloud point of the linear PTEGA was ~70 °C [4]. Thus, the thermosensitive behaviours of POEGA gels can be controlled by manipulating the length of the *oligo*(ethylene glycol) chain. 

Based on their overall properties, the most suitable POEGA gel for a particular application can be selected from these new polymers. For example, to separate oil from an oil–water emulsion, a gel that is hydrophobic in water at higher temperatures (60–80 °C) and hydrophilic at ambient temperatures (20–40 °C) can be chosen. By these criteria, the PTEGA and PDEGA gels are suitable for this purpose; however, the DEGA monomer is not commercially available and is difficult to obtain. Therefore, the PTEGA gel appears to be the most suitable among the four gels for oil adsorption.

**Figure 5:**
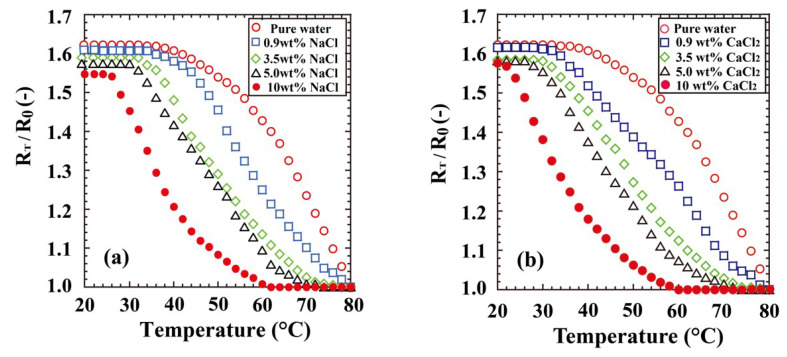
Transition behaviours of PTEGA gel in (**a**) aqueous NaCl and (**b**) CaCl_2_ solutions.

Our previous communication [11] reported the unique transition behaviours of POEGMA_300_ gel in aqueous saline. Although the PNIPA gel hardly swelled in 3.5 wt % aqueous NaCl solution at 20 °C, the POEGMA_300_ gel was well-swollen in a highly concentrated aqueous NaCl solution. Further, the swelling rate and equilibrium swelling ratio were nearly independent of the NaCl concentration at low temperatures, although the equilibrium swelling ratio of the POEGMA_300_ gel was strongly influenced by the NaCl concentration at high temperatures. Figure 5a shows the transition behaviours of the PTEGA gel in pure water and aqueous NaCl solutions of varying concentrations (0.9, 3.5, 5.0, and 10.0 wt %). The transition behaviour of the PTEGA gel responds to the NaCl concentration in a manner similar to that of the POEGMA_300_ and poly(di(ethylene glycol) methyl ether methacrylate) (PDEGMA) gels [11]. In 5 wt % aqueous NaCl solution, the PTEGA gel swells well at 20–30 °C, but *R_T_*/*R*_0_ dramatically decreases for temperatures of 30 °C and higher. The gel does not swell over 70 °C. Poly(ethylene oxide) and its analogues are known to catalyse polymeric phase transfer [39] because they can complex easily with metal ions such as alkali metal or alkaline earth metal ions. In our system, the pendant *oligo*(ethylene glycol) methyl ether chains should similarly form complexes with metal ions. The formation of such complexes causes an increase in the hydrophilicity and the *R_T_*/*R*_0_ of the PTEGA gel, or the salting-in effect, as has been observed previously by Magnusson et al. [10] for copolymers of *oligo*(ethylene glycol) ethyl ether methacrylate and OEGMA, although hydration and swelling of the PTEGA gel are strongly inhibited by the salting-out effect. By these two opposing effects, the *R_T_*/*R*_0_ and LCST values of the PTEGA and POEGMA_300_ gels [11] decrease mildly with increasing NaCl concentration. In addition, the PTEGA gel displays identical swelling behaviours in solutions of NaCl and those of the divalent metal salt CaCl_2_ (Figure 5b). These results suggest that the PTEGA gel has an added advantage over the other gels, because it swells at room temperature and shows excellent thermosensitivity in the presence of highly concentrated metal salts such as NaCl and CaCl_2_. This recommends it as a thermosensitive or absorbent polymer that can be applied in seawater or hard water.

### 3.2. Adsorption of Oil from Oil–Water Emulsion Using PTEGA Gel

The discharge of oil into water and soil is associated with global urbanisation and industrialisation. Oil-contaminated wastewater from oil leaks and industrial discharge causes serious harm to the environment [40,41,42]. Although AC is a popular adsorbent choice, its high production cost and lack of reusability have necessitated the development of substitutes [38]. Synthetic resins could function as effective adsorbents, but their regenerability has not yet been studied [35]. 

**Figure 6:**
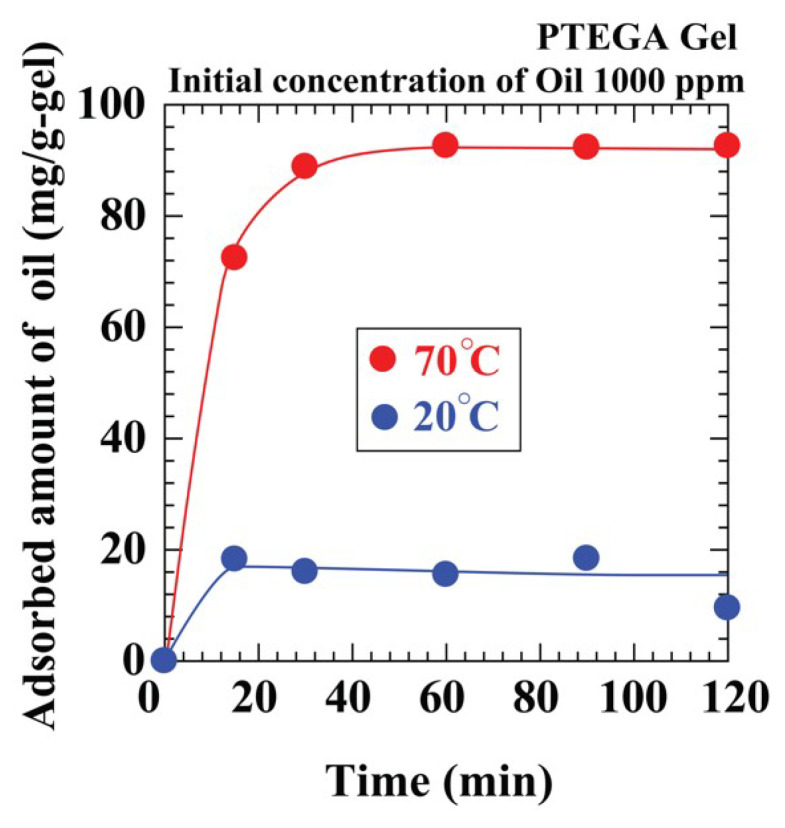
Effect of temperature on oil adsorption amount by PTEGA gel.

Considering these problems, we explored the thermosensitive PTEGA gel as an adsorbent candidate, because its ability to adsorb oil can vary with water temperature. The adsorption system was an oil–water emulsion consisting of *n*-tridecane (1000 ppm) in water at 20 and 70 °C. The results in Figure 6 show that, as the temperature increased, the amount of oil adsorbed increased. At 20 °C, the gel was hydrophilic, and hence, it adsorbed more water than oil and swells. However, the hydrophobicity of the gel increased with increasing temperature. Consequently, the equilibrium capacities at 20 and 70 °C were 16 and 96 mg/g-gel, respectively. When Tween 20 (100 ppm) was added to the system, the equilibrium capacity decreased at higher temperatures (60 mg/g-gel at 70 °C) (Figure S1 in supplementary materials). The equilibrium capacity of AC was 36 mg/g-gel at 70 °C under similar conditions. These capacities of the PTEGA gel remain similar in a 10 wt % aqueous NaCl solution. Therefore, the PTEGA gel is a useful adsorbent in aqueous or saline media at higher temperatures.

**Figure 7:**
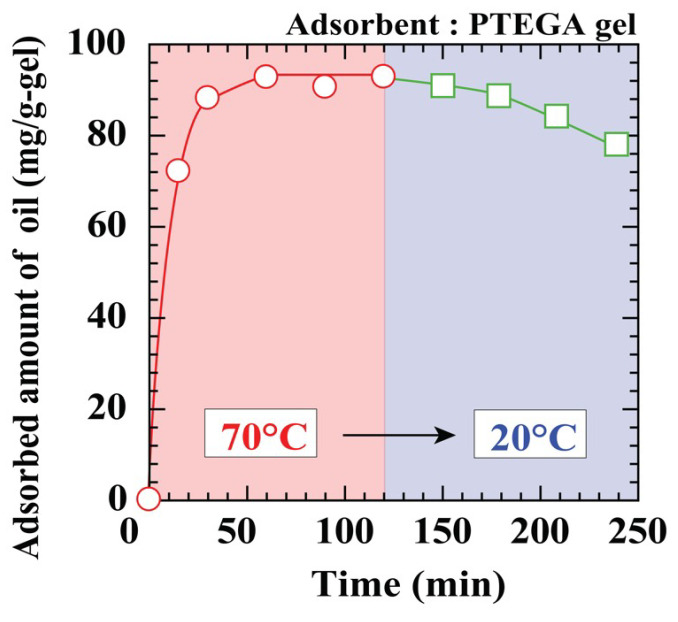
Regeneration of PTEGA gel without surfactant.

Regenerability is one of the most important characteristics for an adsorbent, as it reduces operational costs by allowing repeated reuse [43,44]. The results of the PTEGA gel reusability test for the adsorption and desorption of *n*-tridecane from the oil–water emulsion are shown in Figure 7. The PTEGA gel reaches adsorption equilibrium after 60 min at 70 °C in the absence of Tween 20. Although the fully adsorbed PTEGA gel releases the *n*-tridecane in water with a change in the emulsion temperature from 70 to 20 °C, desorption is extremely slow, and a desorption ratio of ~10 % at 20 °C is reached after 2 h. This may be attributed to the typical non-ionic surfactant structure of the PTEGA gel. The gel functions as a surfactant when the temperature is below the LCST, thus stabilising the oil droplets within the gel and preventing their release. These results suggest that a regeneration system based on the PTEGA gel would be inefficient, despite the release of oil observed at 20 °C. 

## 4. Conclusions

A series of porous POEGA gels with different side chain lengths was obtained via the phase-separation polymerisation of the corresponding monomer solutions in suitable media. The resulting gels rapidly swelled and de-swelled because of their porosities; they showed similar thermosensitive behaviours with different LCSTs. 

The thermal behaviour of the PTEGA gel, representative of the synthesised POEGA gels, was investigated in aqueous NaCl and CaCl_2_ solutions. A unique salting-in effect was observed in the temperature dependence of the equilibrium swelling ratio for this gel. The LCST of the PTEGA gel decreased as the concentration of the aqueous salt solution increased. The gel swelled well at room temperature and showed excellent thermosensitivity in highly concentrated salt solutions. Therefore, POEGA gels can be used as thermosensitive or adsorbent polymers for various purposes in seawater or hard water. We explored the use of the PTEGA gel as an oil adsorbent in an *n*-tridecane-water emulsion. The PTEGA gel showed excellent oil adsorption capacity at 70 °C, with *n*-tridecane adsorption increasing with increasing temperature. However, the release of *n*-tridecane from the fully adsorbed PTEGA gel was very slow, despite the change in water temperature from 70 to 20 °C.

## Figures and Tables

**Table 1:** Preparation of porous thermosensitive gels. ^1^

Gel	Monomer	*R*	*n*	Solvent	LCST^a^ (°C) ^2^
PeDEGA^b^	eDEGA	C_2_H_5_	2	water ^3^ 30 wt % aqueous ethanol	~14 (13) ^4^
PDEGA^c^	DEGA	CH_3_	2	water ^3^ 15 wt % aqueous ethanol	~52 (40) ^4^
PTEGA^d^	TEGA	CH_3_	3	water	~80 (70) ^4^
POEGA^e^_480_	OEGA_480_	CH_3_	8–9	water ^3^ aqueous 5 wt % NaCl solution	>100 (92) ^4^

^a^ Lower Critical Solution Temperature, ^b^ Poly(di(ethylene glycol) monoethyl ether acrylate), ^c^ Poly(di(ethylene glycol) monomethyl ether acrylate), ^d^ Poly(tri(ethylene glycol) monomethyl ether acrylate), ^e^ Poly(*oligo*(ethylene glycol) monomethyl ether acrylate). ¹ Polymerisation of the monomer solution (25 wt %) with DEGDA (2 mol %) in the solvent was performed in the presence of K_2_S_2_O_8_–K_2_SO_3_ as initiators under N_2_ atmosphere at 80 °C for 12 h; ^2^ LCST of the resulting gel was determined from Figure 4; ^3^ a uniform sponge-like gel was not obtained; ^4^ cloud point of the corresponding linear polymer reported by Vancoillie et al. [4].

**Table 2:** Compositions of the oil–water emulsions

	Materials	Quantity
**Oil**	*n*-tridecane	1000 ppm
**Surfactant**	Tween 20	0, 100 ppm
**Salt**	NaCl	0–25 wt %
**Solvent**	distilled water	

## Data Availability

The datasets analysed during the current study are available from the corresponding author on reasonable request.

## Supplementary Materials

The following is available online at https://www.mdpi.com/2073-4360/12/6/1405/s1, Figure S1: Effect of temperature on oil adsorption by PTEGA gel.
