# Supplementary material for: Synthesis, Phase-Transition Behaviour, and Oil Adsorption Performance of Porous Poly(oligo(ethylene glycol) Alkyl Ether Acrylate) Gels
Source: Polymers (Basel). 2020 Jun 23;12(6):1405. doi: 10.3390/polym12061405 (PMC7361678; doi:10.3390/polym12061405)
Supplement: Supplementary file 1 [file polymers-12-01405-s001.pdf]

Supplementary Materials for

Synthesis, phase-transition behaviour, and oil adsorption performance of porous poly(*oligo*(ethylene glycol) alkyl ether acrylate) gels

Syed Ragib Safi, Taku Nakata, Shyotaro Hara, Takehiko Gotoh*, Takashi Iizawa and Satoshi Nakai

Department of Chemical Engineering, Hiroshima University, 1-4-1 Kagamiyama, Higashi Hiroshima, Hiroshima, Japan 739-8527

* Correspondence: tgoto@hiroshima-u.ac.jp; Tel.: +81-82-424-7720

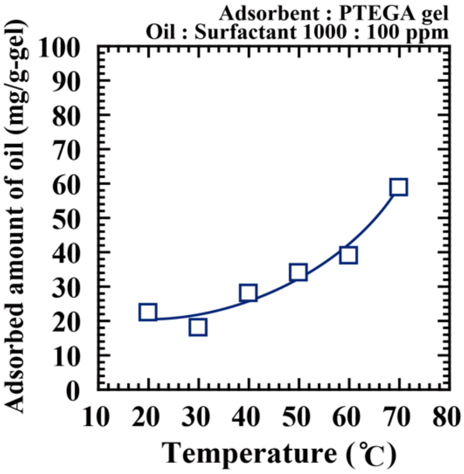

Figure S1. Effect of temperature on oil adsorption by PTEGA gel.
